# Prevalence of mutations in common tumour types in Northern England and comparable utility of national and international Trial Finders

**DOI:** 10.1007/s00432-023-05365-y

**Published:** 2023-09-13

**Authors:** S. Rae, E. Plummer, L. Fitzgerald, L. Hogarth, A. Bridgewood, L. Brown-Schofield, J. Graham, S. Haigh, C. McAnulty, Y. Drew, N. Haris, S. Bashir, R. Plummer, A. Greystoke

**Affiliations:** 1 Sir Bobby Robson Cancer Trials Research Centre, Northern Centre for Cancer Care, Freeman Hospital, Freeman Road, Newcastle upon Tyne, NE7 7DN UK; 2 Newcastle University, Newcastle upon Tyne, NE1 7RU UK; 3 Newcastle Genetics Laboratory, The Newcastle upon Tyne Hospitals NHS Foundation Trust, Newcastle upon Tyne, NE7 7DN UK; 4 BC Cancer Centre, Vancouver, 600W 10th Avenue, Vancouver, BC V5Z 4E6 Canada; 5 University of British Columbia, Vancouver, BC V6T 1Z4 Canada

**Keywords:** Precision oncology, Clinical trials, Trial Finders, ctDNA

## Abstract

**Purpose:**

Tumour genomic profiling is of increasing importance in early phase trials to match patients to targeted therapeutics. Mutations vary by demographic group; however, regional differences are not characterised. This was investigated by comparing mutation prevalence for common cancers presenting to Newcastle Experimental Cancer Medicine Centre (ECMC) to The Cancer Genome Atlas (TCGA) and utility of trial matching modalities.

**Methods:**

Detailed clinicogenomic data were obtained for patients presenting September 2017–December 2020. Prevalence of mutations in lung, colorectal, breast and prostate cancer was compared to TCGA GDC Data Portal. Experimental Cancer (EC) Trial Finder utility in matching trials was compared to a Molecular Tumour Board (MTB) and commercial sequencing reports.

**Results:**

Of 311 patients with advanced cancer, this consisted of lung (*n* = 131, 42.1%), colorectal (*n* = 44, 14.1%), breast (*n* = 36, 11.6%) and prostate (*n* = 18, 5.6%). More than one mutation was identified in the majority (*n* = 260, 84%). Significant prevalence differences compared to TCGA were identified, including a high prevalence of *EGFR* in lung (*P* = 0.001); *RB1* in breast (*P* = 0.0002); and multiple mutations in prostate cancer. EC Trial Finder demonstrated significantly different utility than sequencing reports in identifying trials (*P* = 0.007).

**Conclusions:**

Regional differences in mutations may exist with advanced stage accounting for prevalence of specific mutations. A national Trial Finder shows utility in finding targeted trials whilst commercial sequencing reports may over-report ‘actionable’ mutations. Understanding local prevalence and trial availability could increase enrolment onto matched early phase trials.

**Supplementary Information:**

The online version contains supplementary material available at 10.1007/s00432-023-05365-y.

## Introduction

Our ability to molecularly profile tumours using next-generation sequencing (NGS) has expanded rapidly over the last decade. This is increasingly utilised in early phase clinical trial units to match patients to trials targeting key genomic abnormalities. It is well characterised that using such molecular stratification for early phase trials improves clinical outcomes. (Schwaederle et al. 2015, 2016) This has been further demonstrated in molecular profiling trials aiming to match patients to targeted clinical trials based on mutational profiles. (Stockley et al. 2016; Massard et al. 2017) Classically, this was completed on archival tumour tissue. However, sequencing of circulating tumour DNA (ctDNA), extracted from a ‘liquid biopsy’ blood sample allows for a contemporaneous profile of mutations and provides reliable data, which is increasingly being implemented in the early phase setting. (Adalsteinsson et al. 2017; Merker et al. 2018).

It is recognised that mutations, such as those identified by ctDNA analysis, can vary between demographic groups across multiple solid tumour types (Steuer et al. 2015; Mahal et al. 2020; Yadav et al. 2019), but variations in geographic regions have not yet been well characterised. While it may be supposed that these vary due to interplaying environmental exposures and locoregional resident ethnic populations, this has not been explored in the UK. Newcastle ECMC presents a region suitable for exploration of regional characterisation; with a reasonably static population, which represents the least ethnically diverse in England. (https://www.newcastle.gov.uk/our-city/statistics-and-intelligence, Accessed 14 Feb 2022; https://www.ethnicity-facts-figures.service.gov.uk/uk-population-by-ethnicity/national-and-regional-populations/regional-ethnic-diversity/latest, Accessed 14 Feb 2022) Furthermore, historically associated as an industrial area, with high socioeconomic deprivation and a relatively high smoking prevalence; the North of England represents a base from which to explore the impact of these factors on tumour molecular profiles. (Twigg et al. 2022; https://assets.publishing.service.gov.uk/government/uploads/system/uploads/attachment_data/file/835115/IoD2019_Statistical_Release.pdf, Accessed 12 Apr 2022; Edwards et al. 2006).

Common tumour mutational profiles have been characterised by The Cancer Genome Atlas (TCGA). (https://www.cancer.gov/about-nci/organization/ccg/research/structural-genomics/tcga, Accessed 14 Feb 2022) Over 12 years to 2018, this collated molecular data for over 20,000 primary malignancies across 33 tumour types and provides an online repository (Genomic Data Commons (GDC) Data Portal) for the research community. While limitations exist in tissue biopsy molecular profiling and heterogenous disease stages at sequencing, this provides a valid global comparator against which to explore mutations in multiple tumour types within defined regional populations.

There is no agreed methodology for translating a patient’s profiling results reliably into a targeted trial. Molecular Tumour Boards (MTBs) provide expert opinion in a structured manner, however, are personnel and time intensive and limited by human factors. Likewise, commercial sequencing reports, such as Foundation Liquid® (Roche Group, Basel, Switzerland) the platform utilised in TARGET National (NCT04723316) (Krebs et al. 2016), provide recommendations for trials. However, constructed with an international view these may not provide realistic options at a local centre level, while online resources have been developed to streamline this process such as ONCO KB (www.oncokb.org), My Genome (www.mycancergenome.org) and CIViC (www.civicdb.org)—these remain varyingly accessible to clinicians, non-specific to region and expansive beyond early phase trials and the current clinical realms of molecular analysis.

One solution to these practical challenges of timely patient recruitment lies in the development of free national early clinical trial matching tools, such as the Experimental Cancer Medicine Centre (ECMC) Cancer Research UK (CRUK) Trial Finder—EC Trial Finder (https://www.ecmcnetwork.org.uk/ec-trial-finder, Accessed 14 Feb 2022). This was upgraded in late 2021 and allows for screening using demographic, genomic and geographical information to be input to generate clinical trial recommendations. Access has been rolled out to UK oncologists, potentially representing a means to optimise enrollment to genomically matched trials. To date, real-world utility of EC Trial Finder and comparison with conventional methods of trial identification has not been explored. Here, we investigated regional prevalence of mutations in common tumour types in Northern England and describe the clinical utility of EC Trial Finder compared to commercial reports for this population.

## Methods

Detailed clinicogenomic outcome data were obtained for patients presenting to Newcastle ECMC with tumour profiling completed September 2017–December 2020. Initially, this was mostly composed of archival tumour tissue, but evolved over time to mainly represent ctDNA analysis (Table 1). Prevalence of commonly identified mutations in lung, colorectal, breast and prostate cancer was compared to TCGA GDC Data Portal. EC Trial Finder utility in matching patients to clinical trials was assessed compared to PROSPECT-NE (Molecular PROfiling in Early Clinical Trials—North East) (https://www.hra.nhs.uk/planning-and-improving-research/application-summaries/research-summaries/molecular-profiling-in-early-clinical-trials-north-east-prospect-ne/, Accessed 14 Feb 2022) MTB and sequencing reports. EC Trial Finder is a free online platform open to clinical staff to identify early phase trials open at ECMCs in the UK.

**Table 1:** Profiled patients demographics Northern England September 2017–December 2020

Variable	*n* = 311 (%)
**Sex**	
M	129 (41.5)
F	182 (58.5)
**Age (years)**	
Median (range)	63 (19–97)
**Sequencing method**	
Foundation One® FOL original	151 (48.6)
Foundation One® FOL CDx	80 (25.7)
Qiagen comprehensive cancer panel (PROSPECT-NE)	80 (25.7)
**Tumour profiling**	
Solid tumour tissue	80 (25.7)
ctDNA	231 (74.3)
**Tumour type**	
Lung	131 (42.1)
Colorectal	44 (14.1)
Breast	36 (11.6)
Prostate	18 (5.6)
Pancreatic	12 (3.9)
Cervical	9 (2.9)
Oesophagogastric	8 (2.6)
Ovarian	5 (1.6)
Cancer of unknown primary (CUP)	5 (1.6)
Bladder	5 (1.6)
Other^a^	38 (12.2)

^a^Other—tumour types found in ≤ 4 patients: adrenocortical, appendiceal, cholangiocarcinoma, endometrial, eccrine adenocarcinoma, gastrointestinal stromal cell tumour (GIST), liver, renal, sarcoma, thymic, vulval, no active malignancy

### Sample collection

Informed consent was obtained for collection of blood samples for ctDNA analysis and in the case of PROSPECT-NE, analysis of tumour samples.

### Genomic analysis

The PROSPECT-NE patient cohort had tumour sequencing completed using Qiagen Comprehensive Cancer Panel on an Illumina (San Diego, California, USA) MiSeq at the Northern Genetics Laboratory (Newcastle upon Tyne, UK) with abnormalities identified as significant using SOPHiA GENETICS ® platform (Basel, Switzerland).

ctDNA Sequencing was conducted commercially via a Foundation Medicine (Roche Group, Basel, Switzerland) comprehensive genomic profiling (CGP) NGS ctDNA assay, Foundation One ® Liquid (FOL). Two versions of this assay were utilised, with ‘FOL original’ and an expanded ‘FOL CDx’ implemented from August 2020.

FOL requires two 8.5 ml blood samples to be sent to Foundation Medicine (Massachusetts, USA). Centrifugation is performed to separate plasma from which ctDNA is isolated. NGS of ctDNA is performed using hybridization-based capture technology to analyse 70 (FOL original) or 324 (FOL CDx) genes. FOL detects tumour-related substitutions, insertions, deletions, copy number alterations and gene rearrangements by inputting sequence data into a custom analysis pipeline (www.assets.ctfassets.net). The assay detects alterations in 324 genes. Using the Illumina® HiSeq 4000 platform, hybrid capture-selected libraries are sequenced to uniform depth (targeting > 500X median coverage with > 99% of exons at coverage > 100×).

Details of genomic signature detection are not included in FOL documentation, but Woodhouse et al. outline FOL CDx microsatellite status (MS) and blood tumour mutational burden (bTMB) methods (Woodhouse et al. 2020). For MS, repetitive loci lengths are analysed to detect ‘unstable’ loci. Samples are either deemed Microsatellite Instability (MSI)-high (> 0.5% loci unstable) or MS undetermined. bTMB is determined by dividing the mutation number by the total number of variants counted. Mutations included in the present analysis were deemed to be pathological by the Foundation Medicine assay.

PROSPECT-NE results were reviewed at Newcastle ECMC MTB for clinical significance with appropriate local and national trials identified. Foundation One® reports were reviewed. Abnormalities were recorded as ‘actionable’, and trials recorded as YES if a report suggested a matched trial. Mutations were processed by tumour type using EC Trial Finder (November 2021) and recorded as actionable if trials were suggested. Trials were recorded as YES if open and whether they were ‘all comer’ or recruitment restricted to specific tumour types.

### Statistical analysis

Comparisons between groups were made by the Chi-square test or McNemar’s test as appropriate. Statistical analysis was conducted using SPSS version 27.0 (IBM, New York, NY). *P* values ≤ 0.05 were considered statistically significant. Where applicable Bonferroni correction was applied as corrected *P* value = *P* value * (number of genes in test) ≤ 0.05.

### Ethics statement

This work was under ethics approval from North East–Newcastle & North Tyneside Research Ethics Committee and in the case of PROSPECT-NE patient cohort for participation in a clinical trial matching research study sponsored by The Newcastle upon Tyne Hospitals NHS Foundation Trust and funded by The Newcastle upon Tyne Hospitals NHS Charity.

## Results

### The clinical characteristics of patients and sequencing data

Three hundred and eleven patients with advanced cancer presented to Newcastle ECMC and had tumour profiling completed September 2017–December 2020. Median age was 63 years (range 19–97), with a female predominance (*n* = 182, 58.5%). ‘FOL original’ was the most frequent sequencing technique (*n* = 151, 48.6%), followed by ‘FOL CDx’ and a Qiagen comprehensive cancer panel (PROSPECT-NE), equally (*n* = 80, 25.7%). Tumour types identified at ≥ 5% in the cohort were lung (*n* = 131, 42.1%), colorectal (*n* = 44, 14.1%), breast (*n* = 36, 11.6%) and prostate (*n* = 18, 5.6%) (Table 1). At least one genomic finding was identified in most samples (*n* = 260, 84%).

### Regional prevalence of mutations compared to the Cancer Genome Atlas (TCGA) Genomic Data Commons (GDC) data portal

Significant differences in prevalence of mutations compared to TCGA were identified. Mutations identified at ≥ 5% in the cohort were compared (Table 2). Overall, 1267 lung cancer cases were retrieved from TCGA which revealed a higher relative prevalence of *EGFR* (*n* = 30, 22.9% vs *n* = 148, 11.7%, *P* = 0.001) and *CHEK2* (*n* = 11, 8.4% vs *n* = 20, 1.6%, *P* = 0.000001) mutations in the Northern England population. Six hundred and eleven colorectal cancer cases were retrieved which did not reveal any significantly different prevalence in mutations. 1306 breast cancer cases retrieved also revealed a high relative prevalence of *RB1* (*n* = 5, 13.9% vs *n* = 33, 2.5%, *P* = 0.0002) in the Northern England population. Five hundred and twenty-seven cases of prostate cancer retrieved from TCGA GDC revealed significantly different prevalence of mutations across the majority of mutations identified ≥ 5% including *TP53* (*n* = 13, 72.2% vs *n* = 70, 13.3%, *P* = 0.00001), AR (*n* = 6, 33.3% vs *n* = 4, 0.8%, *P* = 0.00001) and *PTEN* (*n* = 5, 27.8% vs *n* = 19, 3.6%, *P* = 0.00002).

**Table 2:** Mutations in common tumour types identified in Northern England compared to Cancer Genome Atlas

Mutations commonly identified in Newcastle ECMC	Northern England	Cancer Genome Atlas	*P* (Chi-squared)
**Lung**	*n* = 131 (%)	*n* = 1267 (%)	
TP53	72 (55.0)	887 (69.0)	0.11
EGFR	30 (22.9)	148 (11.7)	**0.001**
KRAS	23 (17.6)	208 (16.4)	0.78
RB1	17 (13.0)	82 (6.5)	0.01
STK11	15 (11.5)	117 (9.2)	0.46
ATM	14 (10.7)	104 (8.2)	0.38
CHEK2	11 (8.4)	20 (1.6)	**0.000001**
PIK3CA	11 (8.4)	104 (8.2)	0.95
PTEN	10 (7.6)	89 (7.0)	0.81
DNMT3A	9 (6.9)	44 (3.5)	0.07
**Colorectal**	*n* = 44 (%)	*n* = 610 (%)	
TP53	33 (75.0)	386 (63.3)	0.48
APC	28 (63.6)	486 (79.7)	0.37
KRAS	22 (50.0)	255 (41.8)	0.51
PIK3CA	8 (18.2)	165 (27.0)	0.31
MSH6	3 (6.8)	42 (6.9)	0.77
BRAF	3 (6.8)	86 (14.1)	0.22
NRAS	3 (6.8)	32 (5.2)	0.67
ERBB2	2 (4.5)	39 (6.4)	0.64
**Breast**	*n* = 36 (%)	*n* = 1306 (%)	
TP53	13 (36.1)	473 (36.2)	0.99
PIK3CA	10 (27.8)	435 (33.3)	0.62
RB1	5 (13.9)	33 (2.5)	**0.0002**
PTEN	4 (11.1)	89 (6.8)	0.35
ATM	3 (8.3)	38 (2.9)	0.08
**Prostate**	*n* = 18 (%)	*n* = 527 (%)	
TP53	13 (72.2)	70 (13.3)	**0.00001**
AR	6 (33.3)	4 (0.8)	**0.00001**
PTEN	5 (27.8)	19 (3.6)	**0.00002**
PIK3CA	3 (16.7)	13 (2.5)	**0.00001**
TMPRSS2	3 (16.7)	9 (1.7)	**0.000095**
AKT1	2 (11.1)	3 (0.6)	**0.000013**
CTNNB1	2 (11.1)	12 (2.3)	0.029
NF1	2 (11.1)	2 (0.4)	**0.000001**

Significance has been adjusted using Bonferroni correction

### The utility of Experimental Cancer Medicine Centre (ECMC) Cancer Research UK (CRUK) Trial Finder

**Fig. 1:**
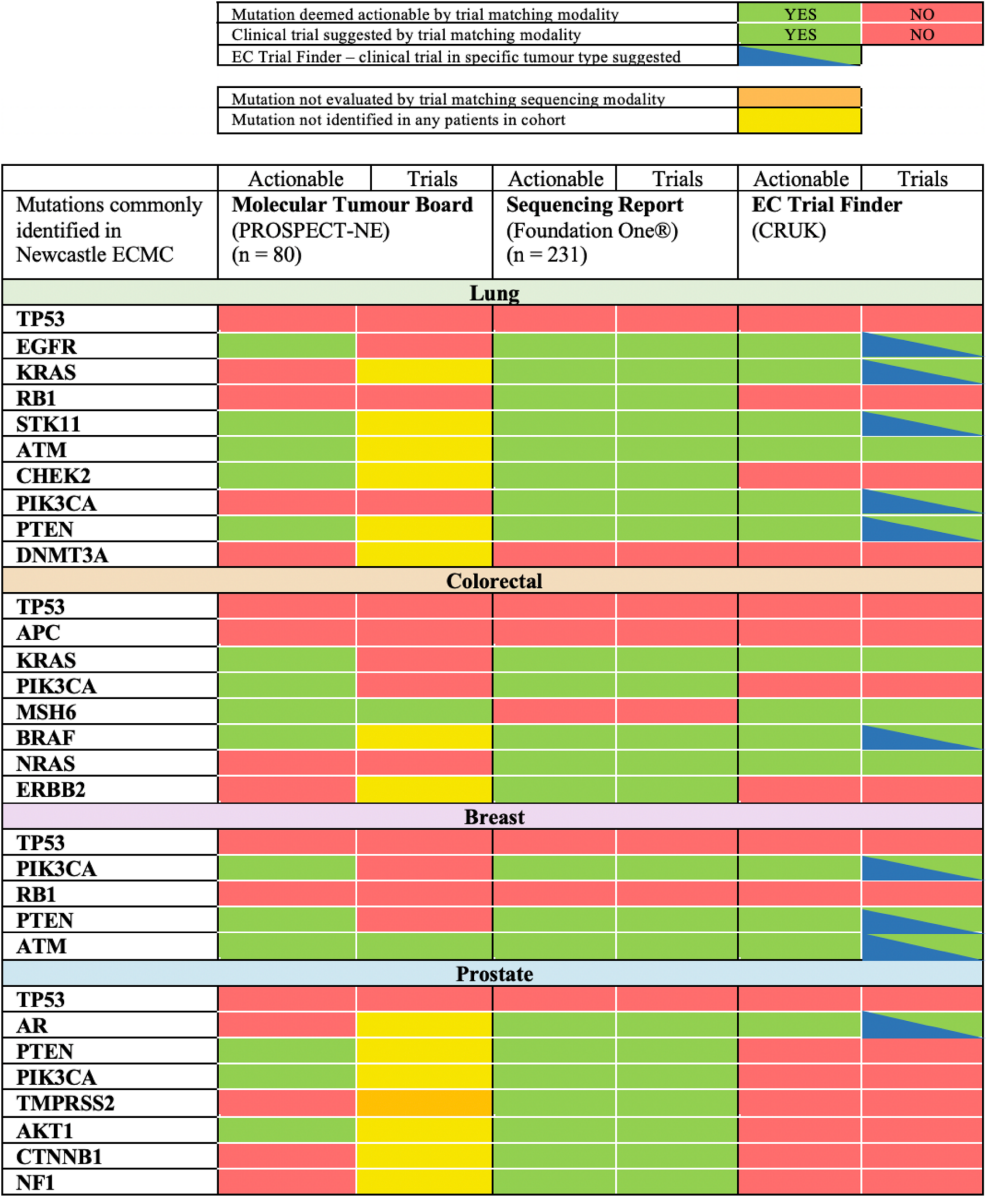
Utility of clinical trial genetic matching modalities in common cancer types and mutations

EC Trial Finder demonstrated significantly different utility than commercial sequencing reports in identifying trials for mutations identified at ≥ 5% prevalence (*P* = 0.007) in lung, colorectal, breast and prostate cancer (Fig. 1 and Appendix 1). Sequencing reports identified actionable mutations and suggested targeted trials in the majority (*n* = 23, 74.1%) of 31 mutations explored across these tumour types compared to less than half (*n* = 14, 45.2%) utilising EC Trial Finder.

Examples of where commercial reports might significantly differ from either the national trial finder or the opinion of the PROSPECT-NE MTB are highlighted by the *CHEK2* abnormalities in 8 patients with lung cancer. These included:

1. Commercial reports described studies which were available in different countries. For example, NCT04123366 was enrolling in Europe but not in the UK and NCT02498613 enrolled from Canada and the USA only. Whilst patient choice is key, there will be additional barriers in patients enrolling on studies in different countries, where they may not be eligible for standard treatment costs. In addition, this may cause harm in terms of financial toxicity and time away from work, family and friends. This was a frequent cause of discrepancy across tumour types.
2. Commercial reports suggested basket studies looking for efficacy through synthetic lethality with either PARP or ATR inhibitors (for example NCT03742895). There is no emerging evidence to support synthetic lethality in this setting, and the identified basket studies were not enrolling patients with those specific genetic abnormalities. This may not be obvious to a commercial company and trial sponsors, as inclusion criteria on clinical trials.gov only state that patients must have mutations in the homologous recombination repair pathway.
3. It was also noted that commercial reports highlighting studies with genomically unselected use of PARP inhibitors. For example, maintenance treatment following first-line treatment with chemotherapy and immunotherapy combinations (NCT03976362). Here, not only are genomic aberrations not a key inclusion criteria, but most patients will not be eligible because of where they are in the disease course in the early phase trials setting, as this is only suitable for previously untreated stage IV patients.
4. Report frequently description studies with the incorrect tumour type. For example, highlighting studies recruiting patients with small-cell lung cancer rather than non-small-cell lung cancer (NCT02769962).

These reasons for discrepancies were not mutually exclusive, and for some studies, more than one reason led to the study not being considered by either the national Trial Finder or the PROSPECT-NE MTB. Similar issues were encountered with other genomics abnormalities and disease types. For example, highlighting studies of PI3 kinase inhibitors in any tumour with an abnormality in a component of this pathway, regardless of the exact gene identified (Appendix 1).

Summary figure of trial exploration for prevalent (≥ 5%) mutations in four most common tumour types. PROSPECT-NE MTB results were retrospectively interrogated using electronic records and classified as potentially actionable by MTB. Potentially targeted trials were recorded. Foundation One® sequencing reports were retrospectively reviewed. Mutations were recorded as actionable, and trials recorded as YES if a report suggested a matched trial. All mutations were processed by tumour type using EC Trial Finder (November 2021) and recorded as actionable if trial modalities were suggested in that tumour type and mutation. Trials were recorded as YES if open and whether they were ‘all comer’ or specific to tumour type. EC Trial Finder demonstrated significantly different utility than sequencing reports in identifying trials for common mutations identified (*P* = 0.007) (McNemar’s). Significance testing criteria were not fulfilled for MTB results.

## Discussion

Profiling of cancer patients’ genomic mutations is an expanding area of research in early phase oncology, with projects such as TARGET National and increased availability of panels such as Foundation One® FOL CDx (Krebs et al. 2016). Clinician understanding in how to respond to and interpret such data, and availability of tools such as trial finders, will define the level of impact this has on real-world patient care, and enrolment into appropriate trials.

In the Northern England cohort, regional differences in genomics were found. This included a relatively high prevalence of epidermal growth factor receptor (EGFR) mutations in lung cancer. EGFR is regarded as ‘actionable’, with oral anticancer therapies such as tyrosine kinase inhibitors established in clinical practice (Tsao et al. 2005; Amalingam et al. 2012; Mok et al. 2017). This was reflected in all matching modalities, with interest continuing in drug development to increase tolerability and target resistance to first-, second- and third-generation *EGFR* targeting inhibitors. In the early phase trial setting, many patients have known EGFR mutations and have progressed on such therapies—making them appropriate candidates for novel trial therapeutics, and potentially enriching the referred population. EGFR mutations are associated with lung cancer in non-smokers despite Northern England representing a high prevalence smoking region (Twigg et al. 2022; Ren et al. 2011).

Checkpoint kinase 2 (CHEK2) mutations were likewise prevalent. This was regarded as potentially actionable at the PROSPECT-NE MTB and on international sequencing report review but not EC Trial Finder—reflecting the demand for an up-to-date national online resource. Unlike EGFR, it does not have approved therapeutics and represents a more challenging and widely regarded as less common mutational profile—representing 1.6% of TCGA cases compared to 8.4% of our lung cohort. Temporaneous discrepancies in findings between modalities may lie in lack of efficacy of targeting CHEK2 having been identified on trial arms and subsequent closures.

In breast cancer, retinoblastoma 1 (RB1) mutations were highly prevalent. It is recognised and reflected in the MTB and EC Trial Finder results that RB1 is challenging to target (Linn et al. 2021). The apparent enrichment of RB1 within the cohort may be accounted for by the advanced disease stage of these patients, as RB1 mutations are recognised as acquired escape mechanisms to CDK4/6 inhibition (Herrera-Abreu et al. 2016).

In prostate cancer, all except one mutation represented significantly different prevalence than TCGA data. Several explanations likely underpin this. Previous cohorts in metastatic castrate resistant prostate cancer (mCRPC) do harbour higher rates of *TP53*, *AR* and *PTEN* mutations compared to TCGA (Robinson et al. 2015). Understanding whether this represents evolutionary changes with progression and standard therapy exposure, or markers of poor prognosis associated with development of metastases remains challenging to determine. Many of the mutations identified in this advanced disease cohort are associated with androgen therapy resistance, reflecting these previous lines of treatment. Furthermore, it might be possible that ctDNA reveals more mutations than metastatic tissue biopsy alone, highlighting advantages of ctDNA for exploring the complete genomic landscape of malignancies with protracted complex courses, such as prostate cancer (Wyatt et al. 2017). Advantages in contemporaneous genomic testing from liquid biopsies compared to archival tissue samples are well characterised and increase the likelihood of clinically meaningful result generation (Hussain et al. 2022). cfDNA sequencing may be better at capturing subclones with particular mutations which were not present in the primary tumour, thus reflecting tumour heterogeneity.

A rationale for increased prevalence of certain mutations in the Northern England population remains beyond the scope of this paper, however, does open interesting avenues in exploration of regional prevalence of mutations and disease biology. With increasing numbers of trial selecting for specific mutations for inclusion and only opening in a limited number of centres, understanding of locoregional centre mutational prevalence could help educated trial location decisions.

Reports such as Foundation One ® detail further information, including bTMB, microsatellite status, and assessment of clonality and germline origin in ctDNA. These may be considered at a local MTB but not always by current online trial finders. Linked interfaces with electronic health records to autopopulate mutational data decrease human error in information throughput and trial recommendations may also prove valuable. Going forward there are both commercial and academic platforms such as eTARGET which are seeking to provide this service (Stevenson et al. 2018).

In recognising the huge potential for a more targeted approach to clinical trials, it is likewise important to acknowledge limitations of this pursuit of precision. In the early phase population, of initially 100 patients, the TARGET National study identified 41 with potentially actionable mutations but yielded partial response in only 4 of 11 matched to targeted trials (Woodhouse et al. 2020). Many so-called umbrella trials such as Lung MATRIX and PRECISION-Panc are now entirely based on streamlining genotype-based treatments either as a pre-requisite for enrollment to an arm or as a means to assess response to treatment (Middleton et al. 2015; Dreyer et al. 2020). It is estimated that around 10% of patients with advanced cancer who undergo genetic profiling will benefit from mutational profile being a factor in their treatment selection (Marquart et al. 2018). For the remaining 90% it is an exciting possibility, that further detailed disease understanding such as gene fusion analysis, RNA sequencing, proteomics and epigenetic status may be included and reveal previously unrecognised driver oncogenic processes beyond somatic DNA mutational assessment (Hilbers 2020; Wheeler et al. 2021; Jordan 2021). Realising an enhanced understanding of tumour profiling, new therapeutics and ultimately processes by which to pair patients to clinical trials will improve precision and outcomes. Presently in the clinic, such mutational data as explored in this work is useful in the identification of a non-actionable mutation, associated with poor prognosis, which may better educate our informed conversations with patients on the realistic outcomes from early phase trials.

This study has limitations in that it is based on data from only around one large UK cancer centre. The method of genomic testing evolved over time with technology advances—from archival tumour sample to cfDNA testing and across an evolution of sequencing panels. It is also noted that the sensitivity of the assays utilised through the TGCA data and the present cohorts differ; however, it is not likely that the differences in mutational prevalence are driven by this with highly significant variances identified. Disease stage matching was not possible due to incomplete data regarding this on TCGA GDC Data Portal. We expect that that the experience we describe may be replicated in many centres and are important factors to consider for the optimal real-world implementation of precision oncology going forward.

## Conclusions

In conclusion, it is necessary to consider our clinical understanding of genomic profiling at both regional and international levels and how we may utilise this knowledge in the early phase trial setting. National clinical trial matching tools such as CRUK EC Trial Finder may enhance our ability to harness these data and match patients with advanced cancer to targeted trials more effectively without identifying trials which are inappropriate or not available in the UK.

## Supplementary Information

Below is the link to the electronic supplementary material.

Supplementary file1 (DOCX 42 KB)

## Data Availability

The datasets generated during and analysed during the current study are available from the corresponding author on reasonable request.
