# Supplementary material for: Prevalence of mutations in common tumour types in Northern England and comparable utility of national and international Trial Finders
Source: J Cancer Res Clin Oncol. 2023 Sep 13;149(18):16355–63. doi: 10.1007/s00432-023-05365-y (PMC10645649; doi:10.1007/s00432-023-05365-y)
Supplement: Supplementary file 1 — Supplementary file1 (DOCX 42 KB) [file 432_2023_5365_MOESM1_ESM.docx]

**Supplementary Appendix | Foundation Medicine Reports Summary**

Summary of Foundation Medicine Reports with apparent discrepancies with results of actionability of tumours from Molecular Tumour Boards and CRUK EC Trial Finder.

| **CHEK2 – Lung Cancer**  (n = 8) | | | | | |
| --- | --- | --- | --- | --- | --- |
| **n Patients** | **Clinical Trial Number** | **Trial Name** | **Locations** | **Targets** | **UK** |
| **8** | **NCT03742895** | **Efficacy and Safety of Olaparib (MK - 7339) in Participants With Previously Treated, Homologous Recombination Repair Mutation (HRRm) or Homologous Recombination Deficiency (HRD) Positive Advanced Cancer (MK - 7339-00 2 / L YNK -002)** | **Newcastle-upon-Tyne (Unit ed Kingdom), Manchester (United Kingdom), Sheffield (United Kingdom), Dublin (Ireland), Oxford (United Kingdom), Cork (Ireland), Villejuif (France), Odense (Denmark), Herlev (Denmark), Copenhagen (Denmark)** | **PARP** | **Yes** |
| **5** | **NCT03976362** | **A Study of Pembrolizumab (MK-3475) With or Without Maintenance Olaparib in First-line Metastatic Squamous Non-small Cell Lung Cancer (NSCLC, MK-7339-008/KEYLYNK 008)** | **Birmingham (United Kingdom), Bury Saint Edmunds (United Kingdom), Colchester (United Kingdom), London (United Kingdom), Swansea (United Kingdom), Westcliff-on-Sea (United Kingdom), Rouen (France), Caen (France), Chauny (France), Essen (Germany)** | **PD-1, PARP** | **Yes** |
| **8** | **NCT02264678** | **Ascending Doses of AZD6738 in Combination With Chemotherapy and/or Novel Anti Cancer Agents** | **Withington (United Kingdom), Cambridge (United Kingdom), London (United Kingdom), Villejuif (France), Saint Herblain (France), Massachusetts, New York, California, Seoul (Korea, Republic of)** | **ATR, PARP, PD-L1** | **Yes** |
| **6** | **NCT03976323** | **Study of Pembrolizumab With Maintenance Olaparib or Maintenance Pemetrexed in First -line (1L) Metastatic Nonsquamous Non-Small-Cell Lung Cancer (NSCLC) (MK-7339-006, KEYLYNK-006)** | **Birmingham (United Kingdom), Bury Saint Edmunds (United Kingdom), Colchester (United Kingdom), London (United Kingdom), Swansea (United Kingdom), Rouen (France), Caen (France), Chauny (France), Essen (Germany), Saint-Mande (France)** | **PARP, PD-1** | **Yes** |
| 8 | NCT04123366 | Study of Olaparib (MK-7339) in Combination With Pembrolizumab (MK-3475) in the Treatment of Homologous Recombination Repair Mutation (HRRm) and/or Homologous Recombination Deficiency (HRD)-Positive Advanced Cancer (MK-7339-007 /KEYL YNK-007) | Rouen (France), La Rochesur Yon (France), Lund (Sweden), Besancon (France), Berlin (Germany), Uppsala (Sweden), Solna (Sweden), Gdansk (Poland), Milano (Italy), Montpellier (France) | PARP, PD-1 | No |
| 8 | NCT03334617 | Phase II Umbrella Study of Novel Anti-cancer Agents in Patients With NSCLC Who Progressed on an Anti-PD- 1/PD-L1 Containing Therapy | Paris (France), Villejuif (France), Nantes Cedex 1 (France), Bordeaux (France), Innsbruck (Austria), Salzburg (Austria), Wien (Austria), Haifa (Israel), Kfar Saba (Israel), Petah Tikva (Israel) | PD-L1, PARP, mTORC1, mTORC2, ATR, CD73, STAT3 | No |
| 7 | NCT02679963 | Trial Evaluating Maintenance Olaparib in Patients With Platinum-sensitive Advanced Non-small Cell Lung Cancer | Villejuif (France) | PARP | No |
| 5 | NCT03297606 | Canadian Profiling and Targeted Agent Utilization Trial (CAPTUR) | Montreal (Canada), Ottawa (Canada), Kingston (Canada), Toronto (Canada), London (Canada), Saskatoon (Canada), Regina (Canada), Edmonton (Canada), Vancouver (Canada) | VEGFRs, ABL, SRC, ALK, AXL, MET, ROS1, TRKA, TRKC, DDR2, KIT, EGFR, PD-L1, CTLA-4, PARP, CDK4, CDK6, CSF1R, FLT3, RET, mTOR, ERBB2, ERBB3, BRAF, MEK, SMO | No |
| 4 | NCT02498613 | A Phase 2 Study of Cediranib in Combination With Olaparib in Advanced Solid Tumors | Massachusetts, Connecticut, Toronto (Canada), Michigan, Virginia, Tennessee, Florida, Texas, California | PARP, VEGFRs | No |
| 3 | NCT02769962 | Trial of CRLX101, a Nanoparticle Camptothecin With Olaparib in People With Relapsed/Refractory Small Cell Lung Cancer | Maryland | PARP, TOP1 | No |
| 1 | NCT03830918 | Niraparib and Temozolomide in Treating Patients With Extensive Stage Small Cell Lung Cancer With a Complete or Partial Response to Platinum-Based First-Line Chemotherapy | California | PARP | No |
| 7 | NCT04276376 | Efficacy and Safety of the Combination of Rucaparib (PARP Inhibitor) and Atezolizumab (Anti-PD-L1 Antibody) in Patients With DNA Repair-deficient or Platinum-sensitive Solid Tumors | Villejuif (France) | PD-L1, PARP | No |
| 3 | NCT03227016 | Study in Patients With SCLC of Veliparib in Combination With Topotecan | München Gauting (Germany) | TOP1, PARP | No |
| 5 | NCT03127215 | Study of Olaparib /Trabectedin vs. Doctor's Choice in Solid Tumors | Heidelber g ( German y ) | FUS-DDIT3, PARP | No |

| **RB1 – Lung Cancer**  (n = 1) | | | | | |
| --- | --- | --- | --- | --- | --- |
| **n Patients** | **Clinical Trial Number** | **Trial Name** | **Locations** | **Targets** | **UK** |
| 1 | NCT02719691 | Phase I Study of MLN0128 and MLN8237 in Patients With Advanced Solid Tumors and Metastatic Triple-negative Breast Cancer | Colorado | Aurora kinase A, mTORC1, mTORC2 | No |
| 1 | NCT03654547 | Safety of TT-00420 Monotherapy in Patients With Advanced Solid Tumors and Triple Negative Breast Cancer | Texas | Aurora kinase A, Aurora kinase B | No |

| **PIK3CA – Breast Cancer**  (n = 8) | | | | | |
| --- | --- | --- | --- | --- | --- |
| **n Patients** | **Clinical Trial Number** | **Trial Name** | **Locations** | **Targets** | **UK** |
| **5** | **NCT03182634** | **The UK Plasma Based Molecular Profiling of Advanced Breast Cancer to Inform Therapeutic CHoices (plasmaMATCH) Trial** | **Multiple UK.** | **ER, EGFR, ERBB2, ERBB4, ATK GFR, PARP** | **Yes** |
| **5** | **NCT03337724** | **A Study of Ipatasertib in Combination With Paclitaxel as a Treatment for Participants With PIK3CA/AKT1/PTEN-Altered, Locally Advanced or Metastatic, Triple-Negative Breast Cancer or Hormone Receptor-Positive, HER2 -Negative Breast Cancer** | **Multiple incl. UK** | **AKTs** | **Yes** |
| **6** | **NCT03424005** | **A Study Evaluating the Efficacy and Safety of Multiple Immunotherapy-Based Treatment Combinations in Patients With Metastatic Triple-Negative Breast Cancer (Morpheus-TNBC)** | **California, Florida, New Jersey, New York, Pennsylvania, Tennessee, Melbourne (Australia), Lyon (France), Toulouse (France), Villejuif CEDEX (France), Erlangen (Germany), Essen (Germany), Seoul (Korea), Barcelona (Spain), Madrid (Spain), Glasgow (United Kingdom), London (United Kingdom)** | **PD-L1, AKT, MEK, VEGF-A** | **Yes** |
| **7** | **NCT03056755** | **Efficacy and Safety of Treatment With Alpelisib Plus Endocrine Therapy in Patients With HR+, HER2-negative aBC, With PIK3CA Mutations, Whose Disease Has Progressed on or After CDK 4/6 Treatment With an Aromatase Inhibit or (AI) or Fulvestrant** | **Multiple incl. UK** | **ER, PI3K-alpha, Aromatase** | **Yes** |
| **4** | **NCT3800836** | **A Study to Evaluate the Safety and Efficacy of Ipatasertib in Combination With Atezolizumab and Paclitaxel or Nab-Paclitaxel in Participants With Locally Advanced or Metastatic Triple-Negative Breast Cancer** | **California, Darlinghurst (Australia), Heidelberg (Australia), Melbourne (Australia), Angers (France), Bordeaux (France), Dijon (France), Paris (France), Villejuif CEDEX (France), Barcelona (Spain), Madrid (Spain), London (United Kingdom), Nottingham (United Kingdom)** | **AKT, PD-L1** | **Yes** |
| **4** | **NCT03395899** | **Pre-operative Immunotherapy Combination Strategies in Breast Cancer** | **London (United Kingdom)** | **MEK, VEGF-A, AKT, PD-L1** | **Yes** |
| **3** | **NCT03673787** | **A Trial of Ipatasertib in Combination With Atezolizumab** | **Sutton (United Kingdom)** | **AKTs, PD-L1** | **Yes** |
| **5** | **NCT03997123** | **Capivasertib+Paclitaxel as First Line Treatment for Patients With Locally Advanced or Metastatic TNBC** | **Multiple incl. UK** | **AKTs** | **Yes** |
| **2** | **NCT03659136** | **The XENERA™- 1 Study Tests Xentuzumab in Combination With Everolimus and Exemestane in Post-menopausal Women With Hormone Receptor Positive and HER2-negative Breast Cancer That Has Spread** | **Multiple incl. UK** | **Aromatase, IGF-1, IGF-2, mTOR** | **Yes** |
| **1** | **NCT02890069** | **A Study of PDR001 in Combination With LCL161, Everolimus or Panobinostat** | **Multiple incl. UK** | **mTOR, PD-1, CXCR2, HDAC, MDM2, IAPs** | **Yes** |
| **3** | **NCT04177108** | **A Study Of Ipatasertib in Combination With Atezolizumab and Paclitaxel as a Treatment for Participants With Locally Advanced or Metastatic Triple-Negative Breast Cancer** | **Multiple UK** | **PD-L1, AKTs** | **Yes** |
| **3** | **NCT04060862** | **A Study of Ipatasertib Plus Palbociclib and Fulvestrant Versus Placebo Plus Palbociclib and Fulvestrant in Hormone** | **Manchester (United Kingdom), London (United Kingdom), Sutton (United Kingdom), Barcelona (Spain), New Jersey, Hamilton (Canada), Georgia, Calgary (Canada), Porto Alegre (Brazil), Malvern (Australia)** | **AKTs, CDK4, CDK6, ER** | **Yes** |
| **3** | **NCT04305496** | **Capivasertib+Fulvestrant vs Placebo+Fulvestrant as Treatment for Locally Advanced (Inoperable) or Metastatic HR+/HER2−Breast Cancer** | **Nottingham (United Kingdom), London (United Kingdom), Paris (France), Villejuif CEDEX (France), Angers (France), Dijon (France), Bordeaux (France), Barcelona (Spain), Madrid (Spain), Sevilla (Spain)** | **AKTs, PD-L1** | **Yes** |
| **1** | **NCT04251533** | **Study Assessing the Efficacy and Safety of Alpelisib + Nab-paclitaxel in Subjects With Advanced TNBC Who Carry Either a PIK3CA Mutation or Have PTEN Loss Without PIK3CA Mutation** | **Nottingham (United Kingdom), Stavanger (Norway), Velbert (Germany), Creteil (France), Reims (France), Angers Cede (France), Metz (France), Saint-Herblain Cédex (France), Oslo (Norway), Poitiers (France)** | **PI3K-alpha** | **Yes** |
| **1** | **NCT03006172** | **To Evaluate the Safety, Tolerability and Pharmacokinetics of GDC-0077 Single Agent in Participants With Solid Tumors and in Combination With Endocrine and Targeted Therapies in Participants With Breast Cancer** | **London (United Kingdom), Surrey (United Kingdom), Villejuif (France), Bordeaux (France), Barcelona (Spain), Valencia (Spain), Massachusetts, New York, Toronto (Canada), Tennessee** | **PI3K-alpha, Aromatase, CDK4, CDK6, ER** | **Yes** |
| 3 | NCT04191499 | A Study Evaluating the Efficacy and Safety of GDC-0077 + Palbociclib + Fulvestrant vs Placebo + Palbociclib + Fulvestrant in Patients With PIK3CA -Mutant, Hormone Receptor-Positive, Her2-Negative, Locally Advanced or Metastatic Breast Cancer | Paderborn (Germany), Saint Petersburg (Russia), Novgorod Veliky (Russia), Moscow (Russia), Yaroslavl (Russia), Volgograd (Russia), Quebec City (Canada), Connecticut, New York, Toronto (Canada) | PI3K-alpha, ER, CCDK4, CDK6 | No |
| 1 | NCT04042051 | Copanlisib in Combination With T-DM1 in Pretreated Unresectable Locally Advanced or Metastatic HER2-positive Breast Cancer | Dublin (Ireland), Cork (Ireland) | PI3K, ERBB2 | No |
| 1 | NCT01805271 | Safety Study of Adding Everolimus to Adjuvant Hormone Therapy in Women With High Risk of Relapse, ER+ and HER2-Primary Breast Cancer, Free of Disease After Receiving at Least One Year of Adjuvant Hormone Therapy | France only | mTOR | No |
| 2 | NCT03386162 | SAFIR PI3K A Phase II Randomized Trial Comparing Alpelisib and Fulvestrant Versus Chemotherapy as Maintenance Therapy in Patients With PIK3CA Mutated Advanced Breast Cancer | France only | PI3K-alpha, ER | No |
| 2 | NCT02299999 | SAFIR0 2_Breast-Efficacy of Genome Analysis as a Therapeutic Decision Tool for Patients With Metastatic Breast Cancer | France only | mTORC1, mTORC2, FGFRs, AKTs, EGFR, ERBB2, ERBB3, MEK, RET, SRC, VEGFRs, AR, PARP, PD-L1 | No |
| 1 | NCT02734615 | Phase I/Ib Trial of LSZ10 2 Single Agent or LSZ102 + LEE011 or LSZ102 + BYL719 in ER+ Breast Cancers | Milano (Italy), Massachusetts, New York, Texas, Kotoku (Japan), Bruxelles (Belgium), Lyon Cedex (France), Singapore | ER, CDK6, CDK4, PI3K-alpha | No |
| 2 | NCT03517956 | Phase 1 Study of the Combination of Rogaratinib With Copanlisib in Patients With Fibroblast Growth Factor Receptor (FGFR)-Positive, Locally Advanced or Metastatic Solid Tumors | California, Illinois, Massachusetts, Michigan, New York, Köln (Germany), Bruxelles (Belgium), Edegem (Belgium), Liege (Belgium), Würzburg (Germany), Seoul (Korea), Singapore, Barcelona (Spain), Valencia (Spain) | FRFG1, FGFR3, FGFR2, FGFR4, PI3K | No |
| 1 | NCT01827384 | Molecular Profiling-Based Targeted Therapy in Treating Patients With Advanced Solid Tumor | USA only | PARP, mTOR, MEK, WEE1 | No |
| 1 | NCT02684032 | A Study To Assess The Tolerability And Clinical Activity Of Gedatolisib In Combination With Palbociclib /Letrozole Or Palbociclib /Fulvestrant In W omen With Metastatic Breast Cancer | USA only | Aromatase, PI3k-alpha, PI3K-gamma, mTORC1, mTORC2, CDK4, CDK6, ER | No |
| 1 | NCT03502733 | Copanlisib and Nivolumab in Treating Patients With Metastatic Solid Tumors or Lymphoma | USA only | PI3K, PD-1 | No |
| 1 | NCT03280563 | A Study of Multiple Immunotherapy Based Treatment Combinations in Hormone Receptor (HR)-Positive Human Epidermal Growth Factor Receptor 2 (HER2)-Negative Breast Cancer | Alabama, California, Maryland, New York, North Carolina, Ohio, Oregon, Pennsylvania, Tennessee, Texas, Ramat Gan (Israel), Tel Aviv (Israel), Gyeonggi-do (Korea), Seoul (Korea) | PD-L1, ER, HDAC, AKTs | No |

| **PIK3CA – Prostate Cancer**  (n = 3) | | | | | |
| --- | --- | --- | --- | --- | --- |
| **n Patients** | **Clinical Trial Number** | **Trial Name** | **Locations** | **Targets** | **UK** |
| **1** | **NCT03673787** | **A Trial of Ipatasertib in Combination With Atezolizumab** | **Sutton (UK)** | **AKTs, PD-L1** | **Yes** |
| **13** | **NCT03006172** | **To Evaluate the Safety, Tolerability, and Pharmacokinetics of GDC-0077 Single Agent in Participants With Solid Tumors and in Combination With Endocrine and Targeted Therapies in Participants With Breast Cancer** | **Multiple incl. UK** | **PI3K-alpha, Aromatase, CDK4, CDK6, ER** | **Yes** |
| 1 | NCT03517956 | Phase 1 Study of the Combination of Rogaratinib With Copanlisibin Patients With Fibroblast Growth Factor Receptor (FGFR)-Positive, Locally Advanced or Metastatic Solid Tumors | Multiple none UK | FGFR1, FGFR2, FGFR3, FGFR4, PI3K | No |
| 1 | NCT03840200 | A Study Evaluating the Safety, Pharmacokinetics and Efficacy of Ipatasertib Administered in Combination With Rucaparib in Participants With Advanced Breast, Ovarian Cancer, and Prostate Cancer | Multiple none UK | PARP, AKTs | No |
| 1 | NCT01827384 | Molecular Profiling-Based Targeted Therapy in Treating Patients With Advanced Solid Tumors | USA only | PARP, mTOR, MEK, WEE1 | No |
| 1 | NCT02565901 | Sirolimus, Docetaxel, and Carboplatin in Treating Patients With Metastatic Hormone-Resistant Prostate Cancer | Washington (USA) only | mTOR | No |
| 1 | NCT03239015 | Efficacy and Safety of Targeted Precision Therapy in Refractory Tumor With Druggable Molecular Event | Shanghai only | EGFR, ERBB2, ERBB4, PARP, mTOR, MET, RET, ROS1, VEGFRs, BRAF, CDK4, CDK6 | No |
| 3 | NCT03297606 | Canadian Profiling and Targeted Agent Utilization Trial (CAPTUR) | Canada only | VEGFRs, ABL, SRC, ALK, AXL, MET, ROS1, TRKA, TRKC, DDR2, KIT, PDGFRs, EGFR, PD-1, CTLA-4, PARP, CDK4, CDK6, CSF1R, FLT3, RET, mTOR, ERBB2, ERBB3, BRAF, MEK, SMO | No |
| 1 | NCT02576444 | OLAParib COmbinations | USA only | PARP, AKTs, WEE`, mTORC1, mTORC2 | No |
| 2 | NCT03502733 | Copanlisib and Nivolumab in Treating Patients With Metastatic Solid Tumors or Lymphoma | USA only | PI3K, PD-1 | No |
| 2 | NCT03994796 | Genetic Testing in Guiding Treatment for Patients With Brain Metastases | USA only | ALK, ROS1, TRKA, TRKB, TRKC, CDK4, CDK6, PI3K, mTOR | No |
| 2 | NCT04253262 | A Study of Copanlisib Combined With Rucaparib in Patients With Metastatic Castration-resistant Prostate Cancer | Rhode Island only | PARP, PI3K | No |
| 1 | NCT01485861 | Study of Ipatasertib or GDC-0980 With Abiraterone Acetate Versus Coralie in Participants With Castration-Resistant Prostate Cancer Previously Treated With Docetaxel Chemotherapy | USA only | AKTs, CYP17 | No |
| 2 | NCT03366103 | Navitoclax and Vistusertib in Treating Patients With Relapsed Small Cell Lung Cancer and Other Solid Tumors | USA only | mTORC1, mTORC2, BCL2, BCL-XL, BCL-W | No |
| 2 | NCT03711058 | Study of PI3Kinase Inhibition (Copanlisib) and Anti-PD-1 Antibody Nivolumab in Relapsed/Refractory Solid Tumors With Expansions in Mismatch-repair Proficient (MSS) Colorectal Cancer | Maryland only | PD-L1, PI3K | No |
| 2 | NCT03190174 | Nivolumab (Opdivo®) Plus ABI-009 (Nab-rapamycin) for Advanced Sarcoma | California only | mTOR, PD-1 | No |
| 2 | NCT02688881 | Study to Evaluate the Safety and Efficacy of Sirolimus, in Subject With Refractory Solid Tumor s | Seoul only | mTOR | No |
| 2 | NCT03842228 | Copanlisib, Olaparib, and Durvalumab in Treating Patients With Metastatic or Unresectable Solid Tumors | USA only | PI3K, PD-L1, PARP | No |

| **TMPRSS2 Fusion – Prostate Cancer**  (n = 2) | | | | | |
| --- | --- | --- | --- | --- | --- |
| **n Patients** | **Clinical Trial Number** | **Trial Name** | **Locations** | **Targets** | **UK** |
| **2** | **NCT03748641** | **A Study of Niraparib in Combination With Abiraterone Acetate and Prednisone Versus Abiraterone Acetate and Prednisone for Treatment of Participants With Metastatic Prostate Cancer** | **Multiple incl. UK** | **CYP17, PARP** | **Yes** |
| **2** | **NCT03395197** | **Talazoparib + Enzalutamide vs. Enzalutamide Monotherapy in mCRPC (TALAPR O-2)** | **Multiple incl. UK** | **PARP** | **Yes** |
| **2** | **NCT03834519** | **Study of Pembrolizumab (MK-3475) Plus Olaparib Versus Abiraterone Acetate or Enzalutamide in Metastatic Castration-resistant Prostate Cancer (mCRPC) (MK-7339010 /KEYLYNK -010)** | **Multiple incl. UK** | **PD-1, CYP17, PARP, AR** | **Yes** |
| **2** | **NCT02264678** | **Ascending Doses of AZD6738 in Combination With Chemotherapy and/or Novel Anti Cancer Agents** | **Multiple incl. UK** | **ATR, PARP, PD-L1** | **Yes** |
| **2** | **NCT02861573** | **Study of Pembrolizumab (MK-3475) Combination Therapies in Metastatic Castration-Resistant Prostate Cancer (MK-3475-365/KEYNOTE -365)** | **Multiple UK** | **AR, PD-1, PARP, CYP17** | **Yes** |
| 2 | NCT04276376 | Efficacy and Safety of the Combination of Rucaparib (PARP Inhibitor) and Atezolizumab (Anti-PD-L1 Antibody) in Patients With DNA Repair-deficient or Platinum-sensitive Solid Tumors | Villejuif only | PD-L1, PARP | No |
| 2 | NCT03317392 | Olaparib and Radium Ra 223 Dichloride in Treating Men With Metastatic Castration-Resistant Prostate Cancer That Has Spread to the Bone | USA only | PARP | No |
| 2 | NCT02769962 | Trial of CRLX101, a Nanoparticle Camptothecin With Olaparib in People With Relapsed/Refractory Small Cell Lung Cancer | Maryland only | PARP, TOP1 | No |
| 1 | NCT03516812 | Testosterone and Olaparib in Treating Patients With Castration-Resistant Prostate Cancer | Washington only | PARP | No |
| 1 | NCT03992131 | A Study to Evaluate Rucaparib in Combination With Other Anticancer Agents in Patients With a Solid Tumor (SEASTAR) | USA only | PARP, FGFRs, VEGFRs, TOP1 | No |
| 1 | NCT04253262 | A Study of Copanlisib Combined With Rucaparib in Patients With Metastatic Castration- resistant Prostate Cancer | Rhode Island only | PARP, PI3K | No |
| 1 | NCT03875313 | Study of CB-839 (Telaglenastat) in Combination With Talazoparib in Patients With Solid Tumors | USA only | GLS, PARP | No |
